# Circulating CD34-positive cells are associated with prolonged time to fracture in people with Duchenne muscular dystrophy on chronic glucocorticoids

**DOI:** 10.1093/jbmr/zjaf041

**Published:** 2025-03-13

**Authors:** Angela Sadlowski, Julia See, Sonum Bharill, Weixin Zhang, Arryn Otte, Emely Loscalzo, Nazanin Yousefzadeh, Ethan Gough, Tricia Nilles, Sisir Barik, Malinda Wu, Janet L Crane

**Affiliations:** Department of Pediatrics, School of Medicine, Johns Hopkins University, Baltimore, MD 2128, United States; Department of Pediatrics, School of Medicine, Johns Hopkins University, Baltimore, MD 2128, United States; Department of Pediatrics, School of Medicine, Johns Hopkins University, Baltimore, MD 2128, United States; Department of Orthopedic Surgery, School of Medicine, Johns Hopkins University, Baltimore, MD 2128, United States; Department of Pediatrics, School of Medicine, Johns Hopkins University, Baltimore, MD 2128, United States; Department of Pediatrics, School of Medicine, Johns Hopkins University, Baltimore, MD 2128, United States; Department of Pediatrics, School of Medicine, Johns Hopkins University, Baltimore, MD 2128, United States; Department of International Health, Bloomberg School of Public Health, Johns Hopkins University, Baltimore, MD 2128, United States; Department of Molecular Microbiology and Immunology, Bloomberg School of Public Health, Johns Hopkins University, Baltimore, MD 2128, United States; Department of Pediatrics, School of Medicine, Johns Hopkins University, Baltimore, MD 2128, United States; Department of Orthopedic Surgery, School of Medicine, Johns Hopkins University, Baltimore, MD 2128, United States; Department of Pediatrics, School of Medicine, Johns Hopkins University, Baltimore, MD 2128, United States; Department of Pediatrics, School of Medicine, Johns Hopkins University, Baltimore, MD 2128, United States; Department of Orthopedic Surgery, School of Medicine, Johns Hopkins University, Baltimore, MD 2128, United States

**Keywords:** osteoporosis, Duchenne muscular dystrophy, biomarker, platelet-derived-growth-factor-type-BB, endothelial cells

## Abstract

Glucocorticoids decrease preosteoclast (POC) platelet-derived-growth-factor-type-BB (PDGF-BB), reducing migration of endothelial and osteo-progenitor cells, impairing skeletal angiogenesis and osteogenesis in mice. To explore human translation, we conducted a case–control study on Duchenne muscular dystrophy (DMD) youth treated with chronic glucocorticoids (n=24) relative to healthy controls (n=13) to explore the association of PDGF-BB, VEGF, angiogenin concentration and peripheral blood mononuclear cell (PBMC) subpopulations as surrogates of POCs (CD14^+^/Stro-1^−^/CD105^−^), skeletal progenitor cells (SPCs: Stro-1^+^/CD105^+^/CD14^−^/CD45^−^), and endothelial/hematopoietic progenitor cells (CD34^+^/CD14^-^/Stro-1^−^/CD105^−^) and CE140b mean fluorescence intensity (MFI) to fracture. People with DMD (8-20 years), were stratified by prior and subsequent fractures relative to biospecimen collection. Healthy controls were age- and sex-matched. Differences between groups were assessed with one-way ANOVA with post-hoc Tukey’s test, retrospective fractures by Kendall Tau correlation, and prospective fractures by bivariable and multivariable accelerated time failure models. Baseline characteristics between groups were similar, though people with DMD were shorter relative to healthy controls, and in the DMD groups, those with prior fractures had a longer duration of glucocorticoid therapy. We noted decreased PDGF-BB concentration and percentages of circulating POCs, SPCs, and CD34+ cells in people with DMD relative to healthy controls. Circulating CD34+ cell percentage positively correlated with PDGF-BB concentration, similar to murine models. Lower percentage of circulating SPCs and CD140b MFI was associated with increased number of retrospective fractures. After a mean follow-up of 2.23 yr, 79% of people with DMD sustained a subsequent fracture. Higher PDGF-BB concentration and percent of POC, SPCs, and CD34+ cells were associated with a longer time to next fracture. After controlling for covariates of fracture risk, increased percentage of CD34+ cells continued to be associated with prolonged time to fracture. Circulating CD34+ cells may thus be a potential biomarker to predict acute fracture risk in young people with DMD on chronic glucocorticoids.

## Introduction

During bone modeling and remodeling, tartrate-resistant acid phosphatase (TRAP)-positive mononuclear cells secrete platelet-derived-growth-factor-type-BB (PDGF-BB) to recruit osteoprogenitor and endothelial cells, which are critical for the acquisition of skeletal mass during growth, maintenance of skeletal mass during adulthood, and fracture healing in mice.^1–3^ Using a young mouse model of glucocorticoid (GC)-induced osteoporosis, we previously reported that GCs inhibit the differentiation of monocytes to TRAP-positive preosteoclasts (POC) and independently suppress *Pdgfb* transcription, resulting in impaired skeletal angiogenesis and osteogenesis.^4^^,^^5^ Chronic GCs have also been reported in mice to suppress vascular endothelial growth factor (VEGF) and angiogenin, leading to endothelial cell senescence, impaired angiogenesis, and subsequent bone loss.^6^ To explore the relevance in humans, we performed a case–control study on a unique population of youth treated with chronic GCs.

Dystrophinopathies, including Duchenne muscular dystrophy (DMD) and Becker MD, are progressive X-linked neuromuscular disorders caused by pathogenic variants in the *dystrophin* gene, impacting the maintenance of the cardiac and pulmonary muscle cell membrane.^7^ Despite the recent approvals of exon skipping and gene editing therapies,^8–11^ GCs remain a mainstay of treatment to prolong ambulation, reduce scoliosis, and improve cardiac and pulmonary function.^7^ GCs have significant endocrine toxicities, most notably on bone health. In childhood, osteoporosis is diagnosed only when there is a significant fracture history.^12^ Using this definition, in people with dystrophinopathies, greater than 75% over the age of 10 years of age have osteoporosis,^13–16^ which can result in loss of ambulation with a lower limb fracture or back pain from a vertebral compression fracture.^17^ A biochemical biomarker associated with fractures could help identify those at highest risk of an acute fracture by allowing the initiation of bone protective interventions earlier.

The invasiveness of transiliac bone biopsies, the gold standard to characterize bone microarchitecture, has precluded in-depth, broad-scale studies in patients with DMD. “Liquid biopsies” that isolate and characterize distinct progenitor cell populations from peripheral blood mononuclear cells (PBMCs) have been validated relative to bone marrow cells in multiple studies in the last 20 years^18–25^ and may offer a substitute to transiliac biopsies. PBMCs that express CD14 can differentiate into osteoclasts in culture and are termed POCs.^22^^,^^26^ Stro-1 and CD105 expressing PBMCs, termed skeletal progenitor cells (SPCs), demonstrate proliferation and osteoblastic differentiation potential.^27^^,^^28^ Numerous cell surface markers have been used to identify and validate circulating endothelial cells isolated from PBMCs; cells expressing CD34 are sensitive, although not specifically restricted to endothelial progenitor cells.^19^^,^^21^^,^^23^^,^^25^^,^^29–32^ We therefore conducted a case–control study of youth with DMD on chronic GCs with and without osteoporosis to determine if there was an association between factors isolated from serum (PDGF-BB, VEGF, and angiogenin) and PBMC subpopulations (POCs, SPCs, and CD34+) cells) with subsequent fractures. Our goal was to identify a biomarker to determine which individuals with DMD on high-dose GCs are at the highest risk of fracture.

## Materials and methods

We conducted a case–control study at the Johns Hopkins University (JHU) and the Kennedy Krieger Institute (KKI) in Baltimore, MD. Pertinent clinical information was collected both retrospectively and prospectively relative to biospecimen collection. JHU and KKI Institutional Review Boards approved this study. Informed written consent/assent was obtained from each research participant and/or their parent if the participant was under 18 years old. Clinical data and a blood sample were collected at the time of enrollment. Clinical information collected included anthropometrics, medications, bone health, endocrine, neuromuscular, cardiac, and pulmonary status.

### Eligibility criteria

Males with confirmed, genetically diagnosed dystrophinopathy aged 8-20 years on chronic GCs were enrolled at JHU and KKI. Research participants were stratified into cases and controls. Cases were defined as research participants with DMD that had a clinically significant fracture history as defined by the International Society of Clinical Densitometry including either a vertebral compression fracture (regardless of BMD Z-score by DXA), two or more long bone fractures by 10 yr of age plus BMD Z-score *<* –2.0, or 3 or more long bone fractures at any age up by 19 yr plus BMD Z-score *<*  –2.0^(^^12^^)^. Disease controls were defined as research participants with DMD who were age-matched within 12 months of cases but did not have a clinically significant fracture history. Healthy controls were also recruited and consented, defined as individuals who were gender- and age-matched within 12 months of cases and did not take any medications for a chronic disease. Exclusion criteria for all groups included metabolic or structural bone diseases other than low bone density, such as hyperparathyroidism, 22q deletion syndrome, celiac disease, osteopetrosis, and McCune-Albright Syndrome. The weight requirement for all subjects was >10 kg due to the volume of blood necessary for collection.

### Clinical records

Retrospective medical records of participants with DMD were collected starting from the initial endocrinology visit. Relevant medical information regarding neuromuscular, cardiac, pulmonary, and endocrine systems was collected. Ambulatory status was assessed using the patient’s description of their daily activities and assessments by a neurologist/physical therapist. Cardiac and pulmonary measures from visits within six months of the endocrine visit were also recorded. Cardiac condition was assessed by a cardiologist based on blood pressure and echocardiogram. Pulmonary data included pulmonary function tests and use of respiratory equipment such as cough assist or bilevel positive airway pressure. Endocrine status included height, weight, pubertal exam (Tanner staging by endocrinologist), fracture history, spine X-rays, and BMD density assessed by DXA. Note that testosterone levels were not used to determine pubertal status, as there is overlap in the normal testosterone ranges at different Tanner staging and would eventually reflect testosterone prescribed for delayed puberty, which afflicts 80% of this cohort.^33^ Medications that could affect bone health, such as testosterone, growth hormone, and bisphosphonate, were also documented with both their dose and frequency.

### Bone health screening

Bone density was measured by DXA (Hologic Inc., Horizon A, S/N 100164). Anterior, middle, and posterior vertebral heights of T4-L4 were measured as previously defined to assess vertebral fractures.^33^ Vertebral fractures were defined as greater than 20% height loss compared to the greatest height of the vertebrae to identify wedge or concave deformities, while the greatest height of the vertebrae immediately above or below was used to identify crush deformities. Long bone fractures were recorded according to patient reports, with qualification of trauma leading to fracture.

### Blood sample collection and biochemical analysis

For each participant, 25 mL of blood was collected in acid citrate dextrose (ACD) vacutainer tubes for PBMC isolation and mixed with an equal volume of Dulbecco’s phosphate-buffered saline (DPBS). Samples were stored at room temperature and processed within 72 hr. PBMCs were separated from red blood cells by Ficoll density gradient centrifugation at 630x g for 25 min. The buffy layer was then isolated and washed with DPBS and spun down. PBMCs were then resuspended in freezing media containing DMSO and frozen in liquid nitrogen. They were then placed in Mr. Frosty/CoolCell at –80 °C for 24 hr and transferred to liquid nitrogen (–180 °C) storage. PBMC isolation generally yielded between 1.5 and 5 × 10^7^ cells with greater than 95% viability. An aliquot of whole blood was frozen from an ACD vacutainer tube. Blood collected in a 5-8 mL serum separator tube was processed within 20-120 min after collection. Serum was isolated by centrifugation at 800x g for 15 min and stored at –80°C in 100 μL aliquots. Serum and whole blood PDGF-BB, VEGF, and angiogenin concentrations were measured by ELISA according to the manufacturer’s protocol (R&D Systems).

### Flow cytometry

Frozen PBMCs were thawed, washed with 1X PBS, and incubated with Zombie NIR Fixable Viability Kit (Biolegend). Cells were pre-incubated with Human TruStain FcX (BioLegend) for 10 min to prevent nonspecific binding. Next, they were incubated for 30 min with an antibody cocktail: PE anti-human CD140b antibody (PDGF receptor beta (PDGFR-β)) (Biolegend, 40:100), PerCP/Cyanine5.5 anti-human CD14 Recombinant Antibody (Biolegend, 5:100), APC anti-Stro-1 Monoclonal Antibody (Invitrogen, 2.5:100), Alexa Fluor 700 anti-human CD45 (Biolegend, 1:100), Brilliant Violet 421 anti-CD105 (Endoglin) (Biolegend, 5:100), and Brilliant Violet 785 anti-human CD34 (Biolegend, 20:100). Then cells were washed with stain buffer (PBS/2% FBS).

The LSRII flow cytometer (BD Biosciences), using FACSDiva software, version 6.13 (BD Biosciences), was used for analysis. FlowJo Software (version 10.2, Tree Star) was used to analyze cell phenotypic data. UltraComp Beads were used for initial compensation (ThermoFisher). Fluorescence-minus-one (FMO), with the omitted antibody’s isotype control in the same concentration, and unstained controls were then used for further compensation. FMOs were made from pooled biospecimen samples. POCs were classified as CD14^+^, Stro-1^−^, and CD105^−^ (Figure S1); SPCs as Stro-1^+^, CD105^+^, CD14^−^, and CD45^−^ (Figure S2) and CD34+ cells as CD34^+^, CD14^−^, Stro-1^−^, and CD105^−^ (Figure S3). CD140b mean fluorescence intensity (MFI) was quantified using FlowJo software. Each subject’s PBMCs were analyzed with at least three distinct runs of 13-25 subjects, with two different samples being run twice to determine intra-assay variability. Experiments were repeated until results could be thoroughly validated in duplicates or triplicates after batch normalization. Results for each subject are presented as the mean across reproducible experiments. Inter- and intra-assay variability values were 9.36% and 9.17%, respectively.

### Statistical analysis

The group sizes (*n*) were 14, 10, and 10 for the cases, DMD controls, and healthy controls, respectively. As this was an initial pilot study, no statistical method was used to predetermine the sample size. Investigators were blinded to allocation during experiments and outcome assessment. All inclusion/exclusion criteria were pre-established, and no samples were excluded from the analysis. For all experiments, *p* <0.05 was considered to be significant. Data are presented as median with either range or 95% confidence interval.

For each specified potential biomarker and CD140b MFI, one-way ANOVA with post-hoc Tukey’s multiple comparisons test was performed on case–control stratification using GraphPad Prism version 9.4.1. Simple linear regression models were used to compare the cell populations (POCs, SPCs, and CD34+ cells) to PDGF-BB concentration as well as to height-adjusted BMD Z-scores (HAZ) using R version 4.2.0 with linear regression models fitted with the pacman package. Spearman correlation was then performed to determine the strength and direction of association between the two variables.

For analysis of retrospective data collected relative to biospecimen collection, we calculated Kendall Tau correlations between each DMD participant’s biomarker value and the number of sustained fractures, as well as their spine deformity index (SDI). SDI was calculated for T4-L4, where each vertebra was assigned a score of 0, 1, 2, or 3 based on severity of compression fracture (none, mild, moderate, or severe, respectively).^33^

For analysis of prospective data collected relative to biospecimen collection, we used accelerated failure time (AFT) regression models for time-to-event data to estimate the relative time to fracture associated with each biomarker, similar to our prior report.^33^ AFT models estimated the relative change in time to fracture per unit of exposure for each risk factor investigated.^34^^,^^35^

Initial AFT results were log transformed such that failure time ratios greater than 0 were protective and failure time ratios less than 0 were detrimental. Repeated observations and recurrent events for participants were included in each model. Robust standard errors were used for statistical inference to account for within-subject correlations. Interval censoring for asymptomatic vertebral fracture time was used to adjust for the ambiguity around the exact timing of vertebral fracture between spine X-rays. Individual risk factors were analyzed using bivariable AFT regression models. Multivariable AFT regression models were fitted to estimate independent associations between each potential biomarker and time to the next fracture. We fitted a separate multivariable AFT model for each biomarker of interest. Covariates were selected based on prior associations with fracture risk and included long bone fracture history, vertebral fracture history, SDI per vertebrae, years of GC use, age at enrollment, height Z-score, BMI Z-score, total body less head (TBLH) BMD HAZ.^7^^,^^13–15^

## Results

### Baseline characteristics

The two control groups, healthy controls and those with DMD without osteoporosis, were age- and sex-matched to cases (DMD with osteoporosis). The DMD group without osteoporosis was the youngest (14.9 yr), but age differences between groups did not reach statistical significance (Table 1). People with DMD, regardless of osteoporosis, were significantly shorter relative to healthy controls (*p* < 0.001 for both post-hoc tests) (Table 1). No statistical difference in weight Z-score or BMI Z-score was found between groups (Table 1). The incidence of vertebral compression fractures was 64% and long bone fractures was 57% in the DMD with osteoporosis group, with the range of age of first fracture occurring between 1.9 and 16.5 yr (Table 2). Per the prespecified stratification, no participants in either control group had a significant fracture history. We analyzed clinical factors potentially associated with fracture risk.^33^^,^^36–38^ Comparing the DMD groups, those with osteoporosis trended toward being on GCs for longer and were more likely not to have begun puberty, although it did not reach statistical significance (Table 1 and Table S1). There was no difference in cardiac or pulmonary complications or ambulatory status in those with DMD (Table S1).

**Table 1:** Baseline characteristics.

	**DMD with osteoporosis**	**DMD without osteoporosis**	**Healthy controls**	***p*-value**
**Demographics**				
**No.**	14	10	10	
**Age (yr), median (range)**	15.8 (11.2, 20.4)	14.9 (9.7, 16.3)	15.3 (11.7, 20.3)	0.61
**Anthropometrics, median (range)**				
**Height (Z-score)**	−3.56 (−5.01, 0.090)	−2.46 (−4.17, −1.24)	0.09 (−1.73, 1.32)	<0.0001
**Weight (Z-score)**	−0.86 (−3.26, 1.86)	−0.25 (−2.02, 2.54)	0.43 (−0.77, 1.28)	0.20
**BMI (Z-score)**	1.23 (−0.88, 2.45)	1.44 (−0.24, 2.63)	0.47 (0.19, 1.53)	0.12
**DXA, median (range)**				
**LS HAZ**	−0.7 (−1.8, 0.4)	−0.1 (−2.3, 1.0)	ND	0.30
**TBLH HAZ**	−2.4 (−4.4, −0.6)	−2.4 (−3.5, −0.9)	ND	0.47
**Glucocorticoid therapy**				
**Yes, *n* (%)**	14 (100)	7 (100)	NA	1
**Age started (yr), median (range)**	4.12 (1.55, 8.17)	5.64 (2.16, 8.85)	NA	0.12
**Duration (yr), median (range) **	11.71 (4.82, 18.82)	8.38 (3.70, 14.04)	NA	0.047
**Daily, *n* (%)**	12 (86)	7 (70)	NA	0.37
**Prednisone, *n* (%)**	1 (7)	0	NA	
**Deflazacort, *n* (%)**	13 (93)	10 (100)	NA	0.41

Abbreviations: DMD, Duchenne muscular dystrophy. HAZ, height-adjusted Z-score; LS, lumbar spine. TBLH, total body less head. NA, not applicable. ND, not determined.

**Table 2:** Fracture history of DMD with osteoporosis.

**Fracture history**	
**Vertebral fractures, *n* (%)**	9 (64)
**No. of Vertebral fractures, median (range)**	2 (0, 3)
**Long bone fractures, *n* (%)**	8 (57)
**Small bone fractures, *n* (%)**	5 (36)
**Age at first fracture (yr), median (range)**	9.53 (1.9, 16.5)
**Time from last long bone fracture (yrs), median (range)**	5.56 (3.42, 9.16)
**Bisphosphonate therapy**	
**Yes, *n* (%)**	12 (85)
**IV**	12 (85)
** Oral**	0 (0)
**Age started (yr), median (range)**	10.26 (4.79, 13.62)
**Duration (yr), median (range)**	5.76 (2.86, 9.49)

### Circulating POC, SPCs, and CD34+ cells are lower in people with DMD on chronic GCs relative to healthy controls

**Figure 1:**
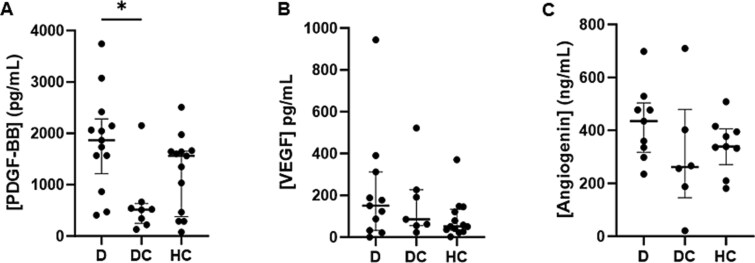
Circulating angiogenic factor concentrations in people with Duchenne muscular dystrophy (DMD) on chronic glucocorticoids with or without fracture relative to healthy controls. (A-C) Platelet-derived growth factor type BB (PDGF-BB) (A), vascular endothelial growth factor (VEGF) (B), and angiogenin (C) were measured by ELISA in whole blood (PDGF-BB) or serum (angiogenin and VEGF) in children and young adults with DMD on chronic glucocorticoids with osteoporosis (D, *n* = 8-11), DMD on chronic steroids without osteoporosis (DC, *n* = 6-8) and healthy controls (HC, *n* = 8-13). Data presented as median with interquartile range. Statistical significance was determined by Kruskal-Wallis test. ^*^p <0.05.

PDGF-BB is secreted from POC to stimulate the migration of osteoprogenitor and endothelial precursor cells to support angiogenesis in mice.^1^ We have previously reported that PDGF-BB, VEGF, and angiogenin concentrations correlate with reduced skeletal angiogenesis in mice exposed to chronic GCs.^6^ Analysis of whole blood and/or serum PDGF-BB, VEGF, and angiogenin concentrations revealed that the median PDGF-BB concentration was not different in those with DMD and osteoporosis relative to healthy controls, but PDGF-BB was significantly decreased in those with DMD without osteoporosis relative to those with osteoporosis (*p* = 0.021) (Figure 1A). No statistically significant differences were noted between the three groups in VEGF and angiogenin concentrations (Figure 1B and C).

**Figure 2:**
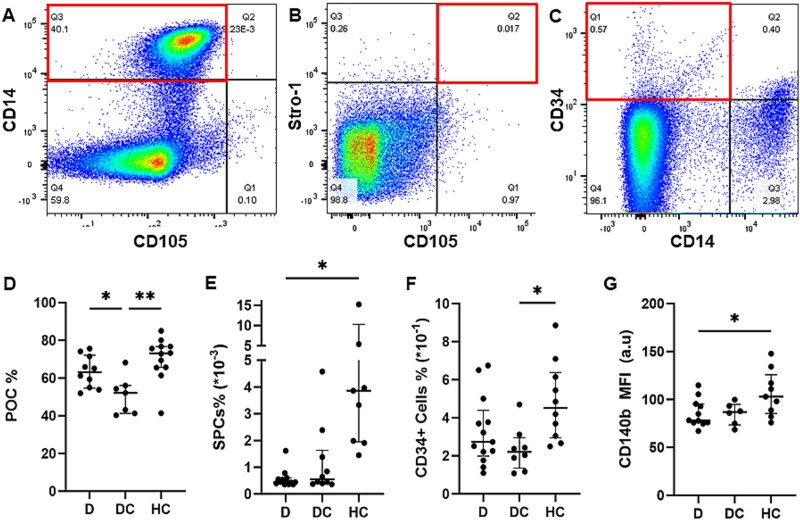
Percentage of circulating preosteoclasts, skeletal progenitors, and circulating CD34+ cells was reduced in people with Duchenne muscular dystrophy (DMD) on chronic glucocorticoids. (A-C) Representative sorting of fluorescence-activated cell sorting by flow cytometry used to identify circulating preosteoclasts (POCs) as CD14^+^/CD105^-^ (A), skeletal progenitor cells (SPCs) as Stro1^+^/CD105^+^ (B), and CD34+ cells as CD34^+^/CD14^-^ (C) in peripheral blood mononuclear cells (PBMCs). (D-F) Quantification of circulating POCs (D), SPCs (E), CD34+ cells (F) in children/young adults with DMD on chronic glucocorticoids with osteoporosis (D), DMD on chronic glucocorticoids without osteoporosis (DC) and healthy controls (HC). (G) Mean fluorescence intensity (MFI) of PDGF receptor beta (CD140b) in all live PBMCs. Data presented as median with interquartile range. *n* = 13-14 in D, 8-10 in DC, and 8-9 in HC. Statistical significance was determined by Kruskal-Wallis test. ^*^p <0.05 ^**^p <0.01.

As bone biopsies are invasive, we evaluated whether precursor cell populations supporting bone remodeling could be identified in the circulation. POCs (CD14^+^Stro1^−^CD105^−^), SPCs (Stro1^+^CD105^+^CD14^−^CD45^−^), and CD34+ cells (CD34^+^CD14^−^Stro1^−^CD105^−^) were isolated from PBMCs using flow cytometry^19–29^ (Figure 2A-C). Surprisingly, the percentage of PBMCs identified as POCs was significantly decreased in those with DMD without osteoporosis relative to both those with osteoporosis (*p* = 0.046) and healthy controls (*p* = 0.0011) (Figure 2D). The percentage of SPCs was significantly decreased in those with DMD and osteoporosis relative to healthy controls (*p* = 0.023) with no statistically significant difference in those with DMD without osteoporosis relative to either group (Figure 2E). The percentage of circulating CD34+ cells was lowest in those with DMD without osteoporosis, reaching statistical significance relative to healthy controls (*p* = 0.0159) but not relative to those with DMD and osteoporosis (Figure 2F).

As PDGF-BB binds to the PDGFR-β, we also analyzed the CD140b (PDGFR-β) MFI in all live cells. We found that those with DMD and osteoporosis had significantly decreased CD140b MFI relative to healthy controls (*p* = .0231) (Figure 2G). The median CD140b MFI was intermediary in those with DMD without osteoporosis, but did not reach statistical significance relative to the other groups (Figure 2G).

### Circulating CD34+ cell percent positively correlates with PDGF-BB concentration, while POC percent negatively correlates with total body, less head BMD height adjusted Z-scores

**Figure 3:**
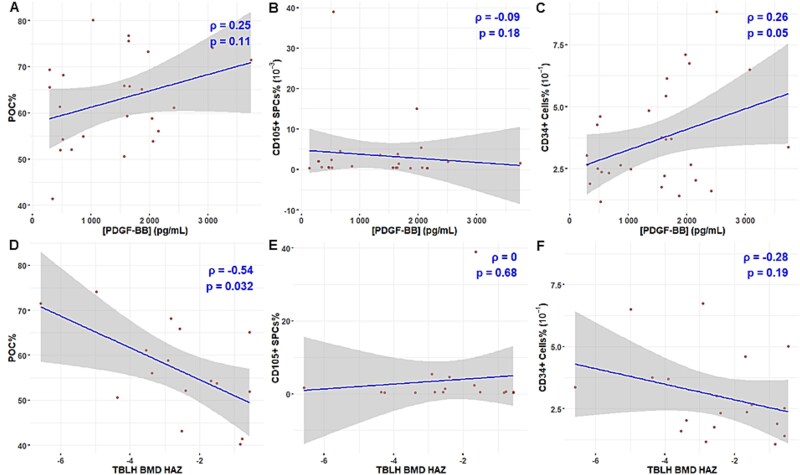
Relationship of circulating preosteoclasts, skeletal progenitor cells, and CD34+ cells to platelet-derived growth factor type BB (PDGF-BB) concentration and total body less head bone mineral density height-adjusted Z-score (TBLH BMD HAZ). (A-C) Simple linear regressions were performed to assess the relationship between whole blood PDGF-BB concentration and percent of circulating preosteoclasts (POC) (A), skeletal progenitor cells (SPCs) (B), and CD34+ cells (C) in children/young adults. n = 23-27. (D-F) Simple linear regressions were performed to assess the relationship between TBLH BMD HAZ and percent of circulating preosteoclasts (POC) (D), skeletal progenitor cells (SPCs) (E), and CD34+ cells (F) in children/young adults with Duchenne muscular dystrophy on chronic glucocorticoids. Statistical significance was determined by Spearman’s rho (ρ) and calculated *p*-value as reported.

To test if a similar relationship is observed between serum PDGF-BB and circulating POCs, SPCs, and CD34+ cells in humans relative to our observations in mice,^1^ we conducted simple linear regression analyses (Figure 3). Linear regression analysis demonstrated a statistically significant positive linear association between PDGF-BB concentration and percentage of circulating CD34+ cells (Spearman’s rho = 0.26, *p* = 0.0499) (Figure 3C). No significant relationship was noted between PDGF-BB concentration and percent of POCs or SPCs. To determine if there was a relationship between circulating precursor cells and the balance of bone modeling/remodeling, we performed simple linear regressions comparing total body less head BMD Z-score after adjusting for age and height^36^ with the circulating precursor cells. Total body less head BMD height-adjusted Z-scores showed a statistically significant negative linear correlation with percentage of POCs (Spearman’s rho = −0.54, *p* = 0.032) (Figure 3D), while no association was noted with SPCs or CD34+ cells.

### Percent of circulating SPCs and PDGFR-β negatively correlates with fracture history

We used Kendall’s Tau to examine the correlation between prior fractures relative to biospecimen collection and PDGF-BB concentration, circulating POC, SPCs, and CD34+ cell PBMC subpopulations, and CD140b MFI (Table 3). The percentage of SPCs was negatively correlated with the number of historical fractures (Kendall’s Tau = –0.39, *p* = 0.010) (Table 3). CD140b (PDGFR-β) MFI negatively correlated with the number of prior fractures (Kendall’s Tau = –0.36, *p* = 0.032). No other biomarker was found to be statistically correlated to the number of prior fractures or severity of vertebral compression fractures as graded by SDI (Table 3).

**Table 3:** Kendall Tau correlation.

	No.	Kendall’s Tau	*p*-value
**Number of fractures prior to biospecimen collection**			
** PDFG-BB**	27	0.28	0.06
** POC**	24	−0.13	0.423
** SPCs**	26	−0.39	0.01
** CD34+ cells**	28	−0.08	0.60
** CD140b MFI**	22	−0.36	0.03
**Spine deformity index at biospecimen collection**			
** PDFG-BB**	17	0.22	0.27
** POC**	16	−0.16	0.44
** SPCs**	17	−0.3	0.14
** CD34+ cells**	18	0.17	0.371
** CD140b MFI**	14	−0.39	0.082

Abbreviations: MFI, mean fluorescence intensity. PDFG-BB, platelet-derived growth factor type BB. POC, preosteoclast. SPC, skeletal progenitor cells.

### Higher percentage of circulating CD34+ cells is associated with a longer time to next fracture

Following biospecimen collection, we prospectively collected clinical data of research participants with DMD for a mean of 2.23 yr (range 5 mo to 4.25 yr). Of the 24 research participants, 19 sustained at least one vertebral compression fracture, and 5 sustained a long bone fracture (Table 4). Based on the original case–control groups, those with osteoporosis at the time of biospecimen collection remained significantly shorter than those who did not have osteoporosis (*p* = 0.037), but no other differences were reported in clinical characteristics (Table 4). Vertebral fractures were noted in 93% of those with osteoporosis and 60% of those without osteoporosis after biospecimen collection (Table 4). In comparison, long bone fractures occurred in 29% of those with osteoporosis and 10% of those without osteoporosis (Table 4).

**Table 4:** Prospective bone health outcomes.

	**DMD with osteoporosis**	**DMD without osteoporosis**	***p*-value**
**Demographics**			
**No.**	14	10	
**Age (yr), median (range)**	18.21 (11.39, 22.42)	16.37 (13.47, 18.01)	0.11
**Length of follow-up (yr), median (range)**	2.38 (1.59, 4.25)	1.92 (1.17, 3.79)	
**Clinical data, median (range)**			
**Height (Z-score)**	−0.74 (−5.10, 0.11)	−1.93 (−2.90, −0.97)	0.037
**Weight (Z-score)**	−0.27 (−3.36, 2.20)	−0.23 (−3.62, 2.08)	0.69
**BMI (Z-score)**	1.51 (−0.86, 2.21)	1.19 (−4.03, 2.48)	0.16
**Fracture history**			
**No. of Participants with vertebral Fracture, *n* (%)**	13 (93)	6 (60)	0.12
**No. of New vertebral fractures, median (range)**	2 (0,6)	2 (1, 3)	0.53
**Long bone fractures, *n* (%)**	4 (29)	1 (10)	0.36
**Small bone fractures, *n* (%)**	(0)	(0)	1
**DXA, median (range)**			
**LS HAZ**	0.1 (−1.7,2.6)	−0.7 (−2.2, 2.1)	0.24
**TBLH HAZ**	−4.3 (−9.4,−1.3)	−3.8 (−9.0, −1.4)	0.92
**Glucocorticoid therapy**			
**Yes, *n* (%)**	14 (100)	9 (90)	
**Age started (yr), median (range) **	4.12 (1.55, 8.17)	5.64 (2.16, 8.85)	0.13
**Duration (yr), median (range) **	13.90 (6.41, 20.41)	10.43 (7.41, 15.66)	0.095
**Daily, median (range) **	11 (79)	7 (70)	0.16
**Prednisone, median (range) **	1 (7)	0 (0)	0.41
**Deflazacort, median (range) **	13 (93)	7 (100)	0.15
**Bisphosphonate therapy**			
**Yes, *n* (%)**	13 (93)	2 (20)	0.25
** IV**	13 (64)	2 (20)	
** Oral**	0 (0)	(0)	
**Age (yr) started, median (range) **	10.26 (4.79, 13.62)	NA	
**Duration (yr), median (range) **	8.10 (5.17, 9.96)	NA	

Abbreviations: DMD, Duchenne muscular dystrophy. HAZ, height-adjusted Z-score. LS, lumbar spine. NA, not available. TBLH, total body less head.

**Figure 4:**
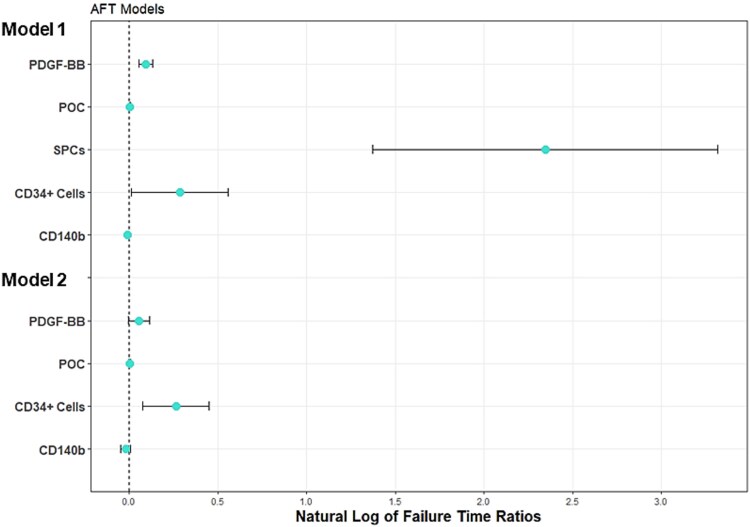
Increased percentage of circulating CD34+ cells was associated with prolonged time to next fracture in children/young adults with Duchenne muscular dystrophy (DMD) on chronic glucocorticoids. Natural log of failure time ratio based on time to next long bone or vertebral compression fracture in children/young adults with DMD on chronic glucocorticoids relative to whole blood platelet-derived-growth-factor-type-BB (PDGF-BB) concentration, circulating preosteoclasts (POC), skeletal progenitor cells (SPCs), and CD34+ cells, and PDGF receptor beta (CD140b) mean fluorescence intensity using a bivariable accelerated failure time analysis (model 1) and a multivariable accelerated failure time analysis (model 2). Covariates included bisphosphonate, growth hormone, and testosterone use, fracture history and severity, glucocorticoid duration, age at enrollment, height Z-score, body mass index Z-score, total body less head bone mineral density height-adjusted Z-score. Data presented as log transformation of failure ratios and 95% confidence interval. Natural log >0 signifies prolonged time to next fracture, < 0 signifies shorter time to next fracture.

We used AFT regression models to determine if the concentration or percentage of the measured biomarkers were associated with time to next fracture. Bivariable AFT linear regression models found that increased concentration of PDGF-BB (*p* < 0.001) and percentage of POC (*p* = 0.001), SPCs (*p* < 0.001), and CD34+ cells (*p* = 0.039) all significantly prolonged time to the next fracture with each having a failure time ratio greater than 0 after a natural log transformation (Figure 4). Conversely, CD140b MFI significantly shortened the time to the next fracture with a failure time ratio less than 0 (*p* < 0.001). Significant covariates that prolonged time to fracture included older age at enrollment, shorter height, prior history of long bone fracture, testosterone usage, further progression through puberty, and being fully ambulatory (Table S2).

We also performed a multivariable analysis to control for covariates potentially associated with fracture risk, including age, fracture history, anthropometrics, BMD, and growth hormone, bisphosphonate, GC, and testosterone use.^33^^,^^36–38^ A higher percentage of circulating CD34+ cells was associated with a statistically significantly increased time to the next fracture (*p* = 0.005). SPCs were excluded from the multivariable analysis, as they were extremely rare in the DMD populations and did not yield enough power to control for the multiple covariates.

## Discussion

In young, growing mice, GCs have been shown to impair POC transcription of PDGF-BB, stimulating the migration of endothelial and osteoprogenitor cells. Therefore, GCs lead to decreased skeletal angiogenesis and osteogenesis. In this study, we noted a similar trend in people with DMD on chronic GCs relative to healthy controls, including reduced concentrations of serum PDGF-BB and percentages of circulating POCs, SPCs, and CD34+ cells in people with DMD treated with chronic GCs relative to healthy controls. Stratifying the people with DMD based on fracture history, we found that children with DMD on chronic GCs with osteoporosis had decreased circulating SPCs and PDGFR-β relative to age- and sex-matched healthy controls. Additionally, we found that children with DMD on chronic GCs without osteoporosis had decreased whole blood PDGF-BB concentrations and circulating POCs relative to those with osteoporosis, along with decreased circulating CD34+ cells relative to age- and sex-matched healthy controls. The majority of people with DMD and osteoporosis were taking bisphosphonates (88%), which should induce osteoclast apoptosis. Zoledronic acid specifically has been found to site-specifically reduce bone vasculature.^39^ Thus, it is possible that bone health interventions may have impacted these factors relative to those with DMD without osteoporosis. Further supporting potential translative meaning, we noted a positive association between PDGF-BB concentration and percent of circulating CD34+ cells and a negative association between BMD and percent of circulating POCs.

People with DMD have a four times higher incidence of all fractures and more than 500-fold higher incidence of vertebral fractures relative to healthy individuals.^33^ Roughly 80% of individuals with DMD will have sustained a fracture by 18 yr of age. In this study, we investigated potential biomarkers for predicting fracture risk. Using Kendall Tau’s test and multilinear accelerated failure time regression models, we found that angiogenic factors and/or circulating precursor cells involved in bone remodeling were associated with fracture risks in an adolescent and young adult population of males with DMD treated with chronic GCs. A lower percentage of circulating SPCs and CD140b (PDGFR-β) MFI was associated with more historical fractures, whereas a higher PDGF-BB concentration and circulating CD34+ cell percentage were associated with a longer time to subsequent fracture. Furthermore, our bivariable AFT analyses of additional covariates known to impact fracture risk (Table S2) validated that the probability of sustaining a fracture was prolonged further in puberty, similar to our previous paper.^33^ We also found that decreasing ambulation was associated with a shorter time to fracture. Other covariates may have been influenced by cofounders such as low BMD by DXA and family history of osteoporosis, which may have potentially led to more aggressive treatment of osteoporosis.

The current clinical care considerations for diagnosing and managing bone health in DMD focus on secondary prevention, noting that BMD Z-score alone, even after adjusting for height and pubertal status, is insufficient to predict fracture risk.^37^ Our study was consistent with this finding, as we did not detect a difference in height-adjusted spine or total body less head Z-scores between patients with DMD with and without osteoporosis. In DMD, each fracture, especially lower extremity fractures, puts patients at increased risk of completely losing ambulation, can result in life-threatening fat emboli, and increases the risk of chronic back pain and lower quality of life.^17^^,^^34^^,^^38^^,^^39^ As such, there is a growing body of research focused on clinical features associated with a higher risk of fracture, including a composite of height Z-score, neuromuscular function, and bone age.^7^^,^^13^^,^^14^^,^^30^^,^^34^ Bone age was not included in our analysis, as few participants had a bone age performed through our routine clinical care. Our study identified several potential biochemical biomarkers measured in the blood, such as PDGF-BB concentration and percentage of circulating POCs and CD34+ cells, that were associated with fracture risk. A blood fracture-risk biomarker would be particularly useful in this population, helping to address the physical limitations some adults with DMD face with the necessary positioning for a DXA scan to monitor bone health accurately. This is becoming more prevalent as people with DMD are now living into their 40s.

There are several limitations of this pilot study. First, the small sample size may have lacked the appropriate power to evaluate all potential biomarkers. For example, PDGF-BB concentration and POC trended toward a positive relationship, which was expected as POC secrete PDGF-BB, but no statistical significance was found (*p* = 0.11). A second limitation is the volume of blood required to isolate and reliably quantitate these rare circulating cells, particularly SPCs. Given the limited number of SPCs detected in people with DMD on chronic GC, we could only analyze SPCs in the univariate model, not the multivariable model. Despite these two limitations, we found that increased serum PDGF-BB and percent of circulating POCs, SPCs, and CD34+ cells were all associated with a longer time to next fracture. This finding aligns with earlier mouse studies showing that PDGF-BB secreted by POCs recruits skeletal and endothelial progenitor cells to the bone remodeling site to couple angiogenesis with osteogenesis.^1^ A third limitation was the inability to internally validate the circulating progenitor cells due to the relative infrequency of circulating SPCs and CD34+ cells. CD34 is expressed by numerous stem progenitor cells, including hematopoietic and endothelial progenitor cells.^31^^,^^40^ CD34^+^/CD45^−^/CD146^+^/CD133^−^ cells are derived from non-hematopoietic lineages and are considered as endothelial colony forming cells or endothelial outgrowth cells, while CD34^+^/CD45^+^/CD133^+^ cells are derived from non-hematopoietic lineages and can differentiate into endothelial cells in culture.^30^ CD34+ cells have been clinically demonstrated to aid in microvascular repair.^30^ Although the absence of CD34 has been traditionally used to identify SPCs,^41^ more recent publications have identified CD34+ cells in the human skeleton in the periosteum, serving as a progenitor population.^42^ Older publications have suggested that CD34+ cells differentiate during fracture repair to aid in both vasculogenesis/angiogenesis and osteogenesis.^32^

Overall, this study had two major findings. First, we validated prior mouse findings regarding the impact of chronic GC exposure on the bone remodeling environment in humans on chronic GCs. Second, we identified potential blood biomarkers associated with time to the next fracture, with circulating CD34+ cells holding the most promise, given the continued association with time to fracture, controlling for covariates. The implications from this study strengthen the validity of the young-GIO mouse model for further mechanistic studies and provide an important translational biomarker for monitoring bone health in children/young adults on chronic GCs. Future prospective longitudinal studies on a larger sample size will be necessary to validate PDGF-BB concentration, circulating POCs and CD34+ cells, and/or CD140b MFI as blood fracture-risk biomarkers, understand how they change over time or with various interventions, and more thoroughly evaluate for additional covariates in this young, vulnerable population to move toward a primary fracture prevention intervention.

## Supplementary Material

Supplemental_Figures_and_Tables_revision_zjaf041

## Data Availability

Data that support the findings of this study are available on request from the corresponding author.
