# Supplementary material for: Circulating CD34-positive cells are associated with prolonged time to fracture in people with Duchenne muscular dystrophy on chronic glucocorticoids
Source: J Bone Miner Res. 2025 Mar 13;40(5):617–27. doi: 10.1093/jbmr/zjaf041 (PMC12103721; doi:10.1093/jbmr/zjaf041)
Supplement: Supplemental_Figures_and_Tables_revision_zjaf041 [file supplemental_figures_and_tables_revision_zjaf041.docx]

**
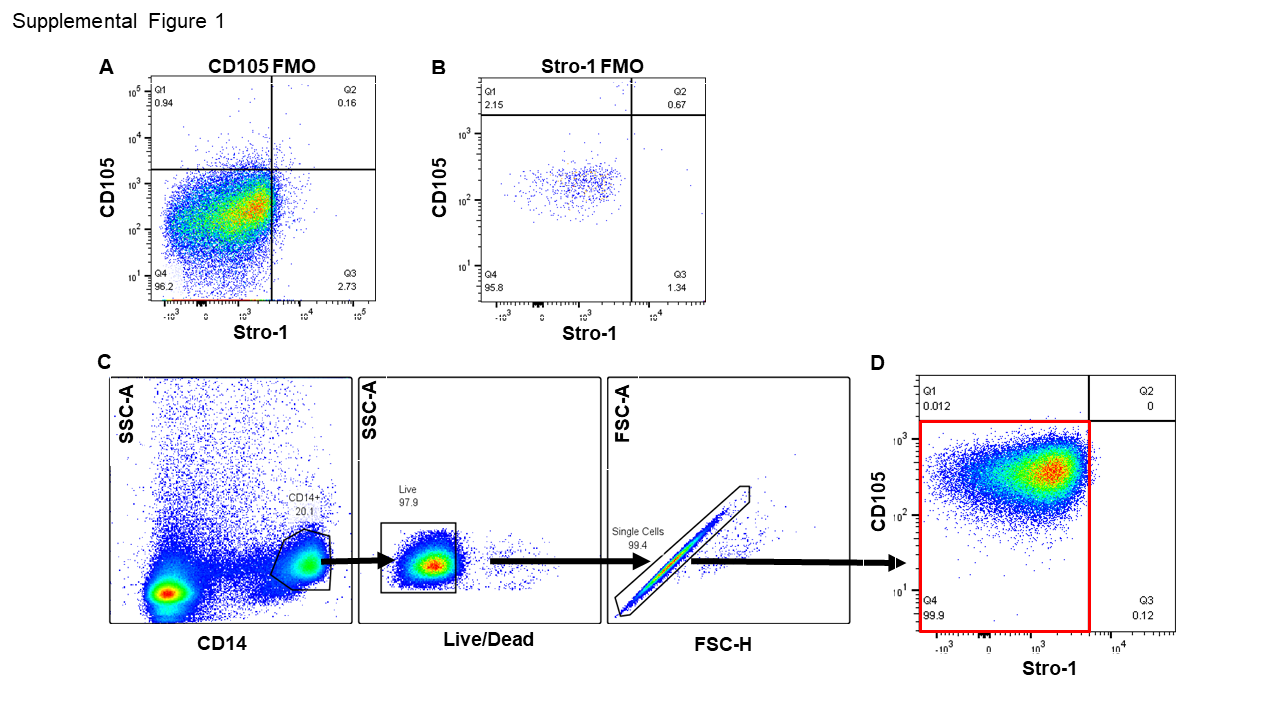
**

**Supplemental Figure 1. Gating strategy and Fluorescence Minus One (FMO) controls for isolating preosteoclasts (POCs) (Stro-1⁻/CD14⁺/CD105⁻) using flow cytometry.** (**A-B**) FMO controls for CD105 (**A**) and Stro-1 (**B**) applied after gating for CD14⁺, viable, and single-cell populations. **(C**) Representative research participant peripheral blood mononuclear cells demonstrating the sequential gating strategy from an independent flow cytometry run with CD14⁺ population identified first versus side scatter (SSC-A), then selected for viability based on APC-Cy7-A Live/Dead staining, which were then selected for single cells using forward scatter area (FSC-A) versus forward scatter height (FSC-H). **(D**) Live, single, CD14+ cells were finally sorted for Stro-1⁻/CD105⁻ based on FMO-defined gating thresholds.


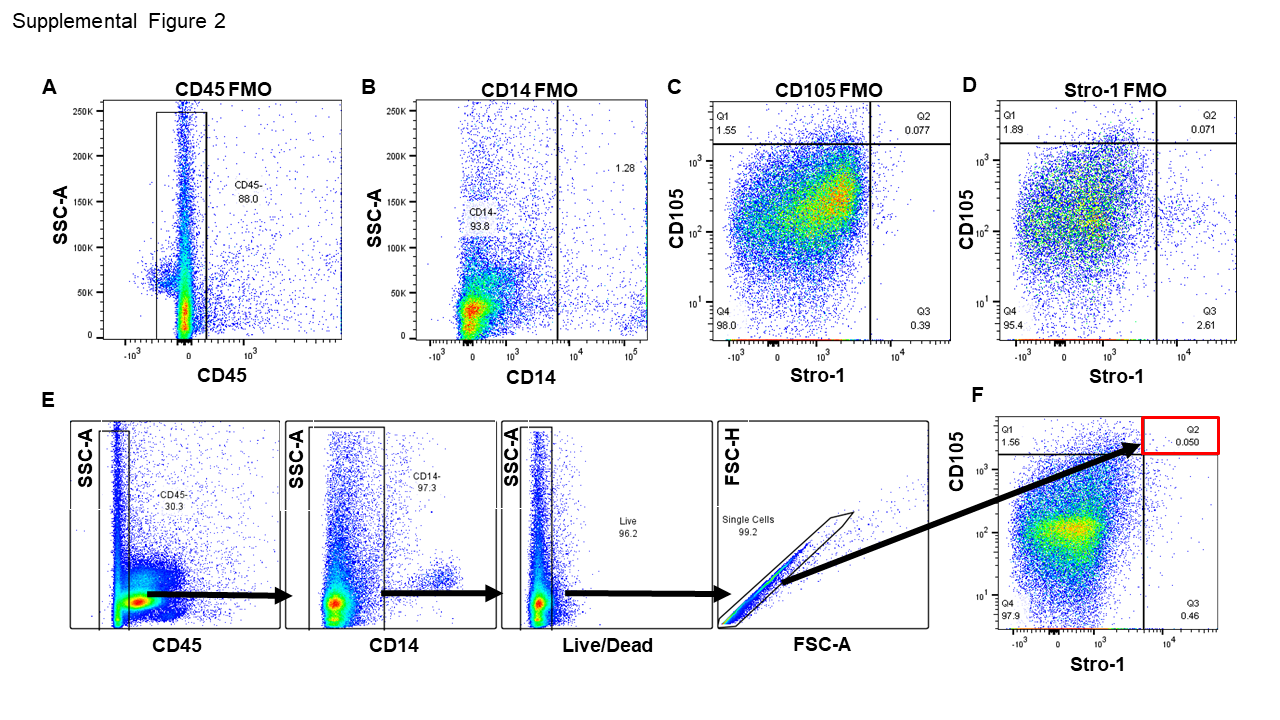


**Supplemental Figure 2. Gating strategy and Fluorescence Minus One (FMO) controls for isolating for Skeletal Progenitor Cells (SPCs) (Stro-1^+^/CD105^+^/CD14^-^/CD45^-^**) **using flow cytometry.** (**A-D**) FMO controls for CD45 (**A**), CD14 applied after gating on the CD45⁻ population (**B**), and CD105 (**C**) and Stro-1 (**D**) applied after gating on the CD45⁻, CD14⁻, viable, and single-cell populations. (**E**) Representative research participant peripheral blood mononuclear cells demonstrating the sequential gating strategy from an independent flow cytometry run with CD45⁻ cells identified first versus side scatter (SSC-A), followed by CD14⁻ cells, then selected for viability based on APC-Cy7-A Live/Dead staining, and then selected for single cells using forward scatter area (FSC-A) versus forward scatter height (FSC-H). (**F**) Live, single, CD45^-^, CD14^-^ cells were finally sorted for Stro-1^+^/CD105^+^ based on FMO-defined gating thresholds.


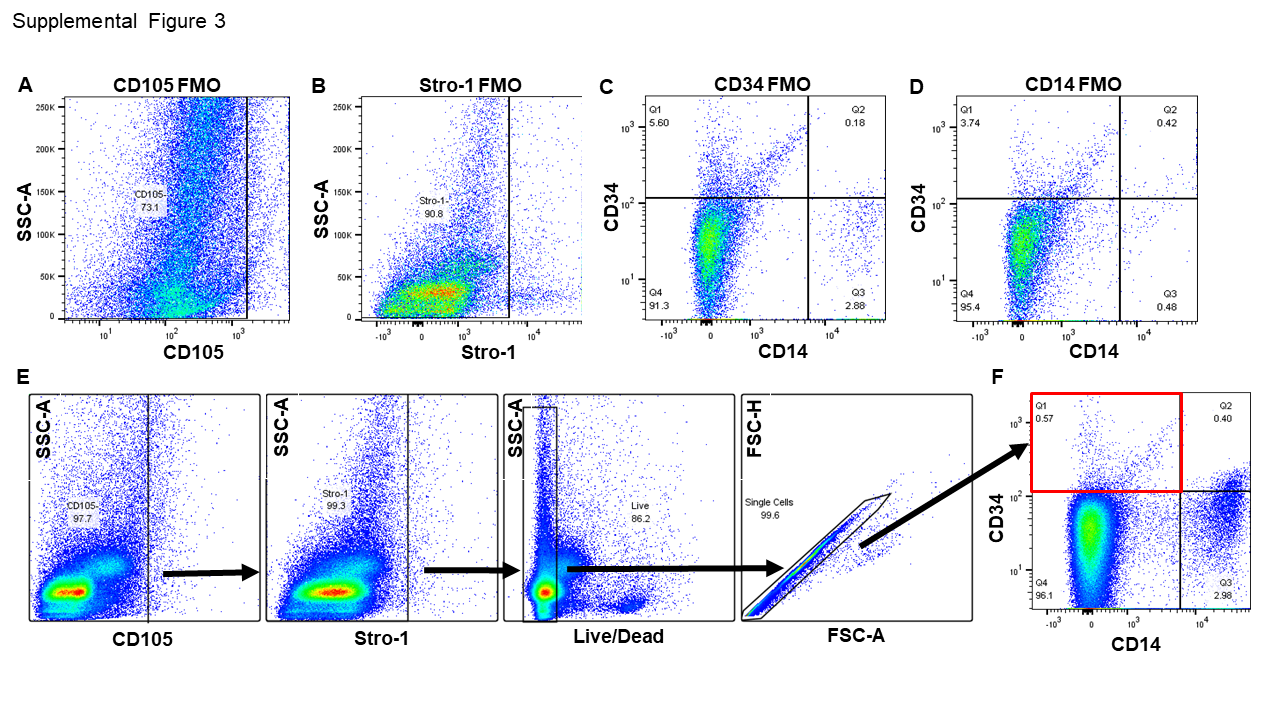

**Supplemental Figure 3. Gating strategy and Fluorescence Minus One (FMO) controls for isolating Circulating CD34+ Cells (CD34^+^/CD14^-^/Stro-1^-^/CD105-) with flow cytometry.** (**A-D**) FMO controls for CD105 (**A**), Stro-1 applied after gating on the CD105⁻ population (**B**), and CD34 (**C**) and CD14 (**D**) applied after gating on the CD105⁻, Stro-1⁻, mononuclear cells demonstrating the sequential gating strategy from an independent flow cytometry run with CD105⁻ cells identified first versus side scatter (SSC-A) followed by Stro-1^-^ cells, then selected for viability based on APC-Cy7-A Live/Dead staining, and then selected for single cells using forward scatter area (FSC-A) versus forward scatter height (FSC-H). (**F**) Live, single, Stro-1^-^, CD105^-^ cells were finally sorted for CD34^+^/CD14^-^ based on FMO-defined thresholds.

| **Supplemental Table 1. Additional Baseline Characteristics** | | | | |
| --- | --- | --- | --- | --- |
|  | | **DMD with Osteoporosis** | **DMD without Osteoporosis** | **p-value** |
|  | | median (range) or N(%) | |  |
| N | | 14 | 10 |  |
| **Ambulatory Status** | | | | |
|  | Fully ambulatory | 1 (7%) | 3(30%) | 0.15 |
|  | Ambulates short distances | 7 (50%) | 3 (30%) | 0.36 |
|  | Unable to ambulate independently | 6 (43%) | 4 (40%) | 0.82 |
| **Pubertal Status** | | | | |
|  | SMR Stage |  |  |  |
|  | 1 | 9 (64%) | 7 (50%) |  |
|  | 2 | 3 (21%) | 2 (20%) |  |
|  | 3 | 1 (7%) | 1 (10%) |  |
|  | 4 | 0 (0%) | 0 (0%) |  |
|  | 5 | 1 (7%) | 0 (0%) |  |
|  | Delayed puberty | 5 (36%) | 3 (30%) | 0.10 |
| **Other pertinent medications** | | | | |
|  | Participation in clinical drug trial | 6 (43%) | 2 (20%) | 0.26 |
|  | Prior growth hormone use | 6 (43%) | 3 (30%) | 0.54 |
|  | Prior testosterone use | 7 (50%) | 2 (20%) | 0.17 |
|  | Current calcium supplementation | 8 (57%) | 4 (40%) | 0.43 |
|  | Current vitamin D supplementation | 13 (93%) | 7 (70%) | 0.15 |
| **Complications** | | | | |
| Cardiac complications | | 14 (100%) | 10(100%) |  |
| Pulmonary complications | |  |  |  |
|  | Use of CPAP and/or cough assist | 4 (29%) | 3 (30%) | 0.94 |
|  | FVC (percent predicted) | 74 (38, 107) | 86 (42, 112) | 0.31 |
|  | FEV1 (percent predicted) | 60 (25, 88) | 74 (29, 95) | 0.33 |
|  | FEV1/FVC | 0.73 (0.58, 0.82) | 0.74 (0.58, 0.88) | 0.92 |
| SMR = sexual maturity rating; CPAP = continuous positive airway pressure; FVC = forced vital capacity; FEV1 = forced expiratory volume in 1 second | | | | |
|  |  |  |  |  |

| **Supplemental Table 2. Bivariable AFT with all the covariates** | | |
| --- | --- | --- |
| **Variable** | **Failure Time Ratio**  **(95%CI)** | **p-value** |
| Years since started GCs | 1.001(0.972,1.032) | 0.932 |
| Enrollment age (per month) | 1.005(1.004,1.005) | **<0.001** |
| BMI Z-score | 1.007(0.960,1.056) | 0.782 |
| Height Z-core | 0.931(0.882,0.983) | **0.01** |
| Total body less head bone mineral density height-adjusted Z-score | 0.964(0.940,0.989) | **0.005** |
| Long bone fracture history (Yes) | 1.163(1.034,1.308) | **0.012** |
| Vitamin D and calcium supplement use (Yes) | 0.880(0.781,0.991) | **0.034** |
| Vertebral fracture history (Yes) | 1.075(0.935,1.238) | 0.31 |
| History of bisphosphonate use (Yes) | 1.111(0.919,1.343) | 0.277 |
| History of growth hormone use (Yes) | 1.037(0.875,1.230) | 0.674 |
| History of testosterone use (Yes) | 1.176(1.014,1.363) | **0.032** |
| Subsequent bisphosphonate use (Yes) | 0.964(0.827,1.124) | 0.642 |
| Subsequent growth hormone use (Yes) | 0.893(0.786,1.013) | 0.078 |
| Subsequent testosterone use (Yes) | 1.121(0.961,1.307) | 0.146 |
| Glucocorticoid use (Yes) | 1.086(0.923,1.277) | 0.32 |
| Family history of osteoporosis (Yes) | 1.207(1.103,1.321) | **<0.001** |
| Tanner stage (3 to 4 relative to 1 to 2) | 1.176(1.032,1.339) | **0.015** |
| **Ambulatory status** |  |  |
| Ambulates short distances (vs full ambulatory) | 0.056(0.024,0.131) | **<0.001** |
| Unable to ambulate independently (vs fully ambulatory) | 0.068(0.029,0.159) | **<0.001** |
